# Biological relevance of ZNF224 expression in chronic lymphocytic leukemia and its implication IN NF-kB pathway regulation

**DOI:** 10.3389/fmolb.2022.1010984

**Published:** 2022-11-09

**Authors:** Rosa Catapano, Leandra Sepe, Elvira Toscano, Giovanni Paolella, Federico Chiurazzi, Serafina Patrizia Barbato, Dario Bruzzese, Rosa Arianna, Michela Grosso, Simona Romano, Maria Fiammetta Romano, Paola Costanzo, Elena Cesaro

**Affiliations:** ^1^ Department of Molecular Medicine and Medical Biotechnology, University of Naples Federico II, Naples, Italy; ^2^ Ceinge Advanced Technologies, Naples, Italy; ^3^ Division of Hematology, Department of Clinical and Experimental Medicine, University of Naples Federico II, Naples, Italy; ^4^ Department of Public Health, University of Naples Federico II, Naples, Italy

**Keywords:** ZNF224, chronic lymphocytic leukemia (CLL), prognostic factor, drug responsiveness, NF-kB pathway, cell survival, cell proliferation

## Abstract

Chronic lymphocytic leukemia (CLL) is a heterogeneous disease, whose presentation and clinical course are highly variable. Identification of novel prognostic factors may contribute to improving the CLL classification and providing indications for treatment options. The zinc finger protein ZNF224 plays a key role in cell transformation, through the control of apoptotic and survival pathways. In this study, we evaluated the potential application of ZNF224 as a novel marker of CLL progression and therapy responsiveness. To this aim, we analyzed ZNF224 expression levels in B lymphocytes from CLL patients at different stages of the disease and in patients showing different treatment outcomes. The expression of ZNF224 was significantly increased in disease progression and dramatically decreased in patients in complete remission after chemotherapy. Gene expression correlation analysis performed on datasets of CLL patients revealed that ZNF224 expression was well correlated with that of some prognostic and predictive markers. Moreover, bioinformatic analysis coupled ZNF224 to NF-κB pathway, and experimental data demonstrated that RNA interference of ZNF224 reduced the activity of the NF-κB survival pathway in CLL cells. Consistently with a pro-survival role, ZNF224 knockdown raised spontaneous and drug-induced apoptosis and inhibited the proliferation of peripheral blood mononuclear cells from CLL patients. Our findings provide evidence for the involvement of ZNF224 in the survival of CLL cells *via* NF-κB pathway modulation, and also suggest ZNF224 as a prognostic and predictive molecular marker of CLL disease.

## Introduction

Chronic lymphocytic leukemia (CLL), the most common type of leukemia in adults, is a malignant disorder of mature B-cells characterized by the clonal proliferation and accumulation of neoplastic lymphocytes in the blood, lymphoid tissue, and bone marrow (Chiorazzi et al., 2005; Kipps et al., 2017). The presentation and clinical course of CLL are highly variable. In some cases, the disease has an indolent course and patients survive for several decades never requiring treatment; in other cases, the disease progresses rapidly and has a poor prognosis (Strati and Shanafelt, 2015; Burger, 2020; Hallek and Al Sawaf, 2021).

In the past few years, therapy for CLL has undergone notable improvements, through the use of novel drugs for patients with refractory CLL (Hillmen, 2011). However, despite these therapeutic advances, CLL remains an incurable disease. Often the patients relapse during treatment and the disease evolves into a more aggressive stage, probably due to the onset of new mutations acquired during the treatment (Maddocks et al., 2015). Microenvironment signals, karyotypic complexity and gene-expression profiling affect survival and proliferation of CLL cells and have a significant impact on the escape of CLL cells from the action of drugs (Burger, 2020). Consequently, early identification of genetic lesions and/or differential expression profile of genes potentially relevant for disease pathogenesis are essential in risk-stratification of CLL patients and in the selection of the best therapeutic strategy to prevent or overcome the onset of drug resistance (Rossi et al., 2017). Clinical staging systems, such as the Rai (Rai et al., 1975) or Binet (Binet et al., 1981), for prognostic assessment of patients with CLL often are not enough in the prediction of disease outcome. To facilitate the identification of patients with poor prognoses and to improve the clinical management of B-CLL, some molecular prognostic markers have been identified over the past decades (Shanafelt et al., 2004; Rossi et al., 2017). Among them, the immunoglobulin heavy-chain gene mutation status (IgV H) (Damle et al., 1999; Hamblin et al., 1999), leukemia-cell expression of tyrosine kinase ZAP-70, antigen CD38 (Damle et al., 1999; Crespo et al., 2003) and cytogenetic aberrations (Baliakas et al., 2014) are regarded as the most reliable for predicting the clinical course of the disease. Deletions and mutations of the TP53 gene belong to the strongest prognostic and predictive markers for therapy response in CLL (Zenz et al., 2010; Rossi et al., 2014). Although in recent years other potential biomarkers have been proposed, such as lipoprotein lipase (LPL) (Van Bockstaele et al., 2007; Mansouri et al., 2010), T-cell leukemia 1 (TCL1) (Browning et al., 2007), Myeloid cell factor-1 (MCL1) (Pepper et al., 2008), discoidin domain receptor 1 (DDR1) (Barisione et al., 2017), the progress in the understanding the distinctive biological characteristics of CLL and the development of novel targeted agents require the identification of new and easy assessable molecular markers that can be added to those already identified, to improve the prediction of disease progression and therapy responsiveness (Butler and Gribben, 2010; Delgado et al., 2020).

The zinc-finger transcription factor ZNF224 plays a pivotal and dual role in human cancer (Cesaro et al., 2017; Sobocińska et al., 2021) influencing pathways associated with cell survival, tumor growth, and apoptosis resistance through the interaction with its cofactors (Di Caprio et al., 2015; Cesaro et al., 2021a).

In particular, in chronic myeloid leukemia, where ZNF224 gene expression is negatively regulated by the Bcr-Abl oncoprotein, we demonstrated that induction of its expression by targeting signaling pathways downstream of Bcr-Abl such as PI3K and Jak/STAT plays a role in overcoming imatinib resistance (Montano et al., 2015; Sodaro et al., 2018a; Sodaro et al., 2018b).

On the other hand, high ZNF224 expression is associated with tumorigenesis in various solid and hematological malignancies. Indeed, ZNF224 plays a relevant role in bladder carcinogenesis causing, through the interaction with the corepressor DEPDC1, the transcriptional inhibition of A20 gene and consequently activating the NF-kB antiapoptotic pathway (Harada et al., 2010).

In CLL, the aberrant ZNF224 expression contributed to apoptosis resistance and impaired proliferation of leukemia cell lines (Busiello et al., 2017).

Recently, it was demonstrated that ZNF224 is involved in the regulation of the TGF-β pathway in melanoma and acts as a mediator of the pro-oncogenic functions of TGF- β sustaining the acquisition of a metastatic phenotype (Cesaro et al., 2021b).

In this study, we aimed to broaden our knowledge of ZNF224 role in CLL progression and clarify the molecular mechanisms by which ZNF224 contributes to the aggressive behavior of this disease.

To assess a potential role of this transcription factor as a novel marker of disease progression and therapy responsiveness in CLL, we measured ZNF224 mRNA levels in B-CLL specimens from patients at different stages of the disease and in a subgroup of patients under treatment. Also, we interrogated datasets publicly available through NCBI Gene Expression Omnibus repository (http://www.ncbi.nlm.nih.gov/geo) to evaluate the correlation between ZNF224 and some recently proposed CLL markers. Moreover, we investigated the effect of ZNF224 downmodulation on spontaneous and drug-induced apoptosis and proliferation of CLL cells. Finally, the same datasets were analyzed to explore mechanisms underlying the pathogenic role of ZNF224 in CLL. The *in silico* data, confirmed by *in vitro* experiments, assigned a role to ZNF224 in the aberrant activation of NF-κB signaling pathway that promotes inappropriate lymphocyte survival, acquisition of resistance to chemotherapy, and deregulated proliferation (Lopez-Guerra and Colomer, 2010; Mansouri et al., 2016).

## Materials and methods

### Peripheral blood samples

Peripheral blood samples of CLL patients were collected at the Division of Hematology, Department of Clinical and Experimental Medicine, University of Naples Federico II. B-CLL diagnosis was obtained according to clinical and immunophenotypic criteria. Peripheral blood samples of healty donors were collected at the Transfusional Centre, University of Naples Federico II. The patients provided appropriate written informed consent according to the guidelines of the Medical Ethical Committee of the University Federico II of Naples, in accordance with Declaration of Helsinki protocols. Clinical informations of patients are summarized in Supplementary Table S1. B-cells were isolated from whole blood by negative selection using the RosetteSep Human B cell enrichment kit (Stem cell Technologies, Vancouver, BC, Canada#15024). Peripheral Blood Mononuclear Cells (PBMCs) were isolated by a Ficoll-PaqueTM density gradient (Merck, Darmstadt, Germany) from the heparinized peripheral blood of B-CLL of untreated patients in the stationary phase of the disease for *in vitro* silencing of ZNF224. CLL patients who had ≥80% CD19^+^/CD5+ co-espressing cells were included in this study (Cesaro et al., 2022). CLL PBMCs were cultured in RPMI 1640 (Sigma-Aldrich, Milan, Italy) supplemented with 10% human serum and stimulating anti-Human CD3 (10μg/1*10^6^ cells) (eBioscience Thermo Fisher, Inc, Waltham, MA) at 37°C in a humidified 5% CO_2_ atmosphere (Romano et al., 2008).

### Transient transfection and cell treatment

For ZNF224 knockdown, 1*10^6^ CLL PBMCs were transiently transfected with 25 pmol of Dharmacon™ ON-TARGETplus HumanZNF224 siRNA-SMARTpool or a non-targeting pool as a control, using Lipofectamine 2000 (Invitrogen, Carlsbad, CA) as transfection reagent. For drugs treatment, 72 h after transfection PBMCs were treated with 1 μM Fludarabine (Teva Pharmaceutical Industries Ltd, UK) or 10 μg/ml Rituximab (Sanzoz GmbH, Kundl, Austria) for 48 h. For the evaluation of ZNF224 mRNA levels, a well of transfected PBMCs, from each experimental point, was collected for RNA isolation and subsequent Real-Time PCR.

### RNA isolation, reverse transcription and real-time PCR

Total RNA was isolated from PBMC or B-cells of CLL patients with Quick-RNA™ MiniPrep Plus (Zymo research, Irvine, CA, United States) according to the manufacturer’s protocol. RNA was reverse-transcribed, and amplified by quantitative Real-time PCR as previously described (Busiello et al., 2017). Specific primers sequences used for ZNF224 and HPRT mRNA amplification were previously reported (Busiello et al., 2017).

The sequences of the other used primers are:

• TNF-a Fw 5′-AGCCCATGTTGTAGCAAACC-3′
• TNF-a Rev 5′-TGAGGTACAGGCCCTCTGAT-3′
• p65 Fw 5′-TATCAGTCAGCGCATCCAGAC-3′
• p65 Rev 5′-ACAGTAGGAAGATCTCATCCC-3′
• p50 Fw 5′-AGCGCCATCTCACTGCTGTG-3′
• p50 Rev 5′-GCTTGAGTAAGATACTGAGAAC-3′
• cyclin D1 Fw 5′-TGTGAAGTTCATTTCCAATCCG-3′
• cyclin D1 Rev 5′-CTTCGATCTGCTCCTGGCAG-3′
• Bcl-2 Fw 5′-CTGCACCTGACGCCCTTCACC-3′
• Bcl-2 Rev 5′-CACATGACCCCACCGAACTCAAAGA-3′
• BAX Fw 5′-TCAGGATGCGTCCACCAAGAAG-3′
• BAX Rev 5′-GCAAAGTAGAAAAGGGCGACAACC-3′

The relative quantification in gene expression was determined using CFX Manager 3.0 software (Bio-Rad Laboratories).

### Cell lysates and western blot assays

Total cell lysates were prepared by homogenization in modified RIPA buffer (Thermo Scientific) supplemented with protease and phosphatase inhibitors, as previously described (Castaldo et al., 2019). The membranes were incubated overnight with the following antibodies: Phospho-NF-κB p65 (Ser536) (93H1) (1:1000 dilution; Cell Signaling, Danvers, MA, United States #3033), NFκB p65 (C-20) (1:300 dilution; Santa Cruz Biotechnology, Santa Cruz, CA, United States #sc-372), IKKα/β and IKKγ (1:1000 dilution; Santa Cruz Biotechnology, #sc-7607 and #sc-8032, respectively), anti-ZNF224 (T3) (Cesaro et al., 2021b) and anti-GAPDH (1:1000 dilution; Cell Signaling #2118). The blots were visualized using Clarity Western ECL Substrate Kit (Bio-Rad Laboratories) and immunoreactive bands were detected by autoradiography or by ChemiDoc XRS Image System (Bio-Rad Laboratories). Quantification of protein bands was obtained by densitometric analysis using the ImageJ software.

### Flow cytometric analysis

Cell death analysis was conducted by Annexin-V (FITC-conjugated, Immunotools) staining, as previously described (Parisi et al., 2017). Briefly, the cells were harvested, washed in PBS, and then incubated for 20 min in the dark with 50 μL of binding buffer containing 1 μL of Annexin-V-FITC (FITC Annexin V poptosis Detection Kit, BD Pharmingen™). Also, to monitor proliferation, after 72 h of transfection with ZNF224 siRNA or scrambled siRNA, PBMC_S_ were collected for nuclear staining of Ki67 protein. To make the nuclei accessible to Ki67 APC-coniugated antibody (Invitrogen™, Thermo Fisher Scientific), cell fixation and permeabilization were performed using the Foxp3/Transcription Factor Staining Buffer Set (eBioscience™, Thermo Fisher Scientific) according to the manufacturer’s protocol. Relative IgG isotype APC-conjugated was used as a control of a non-specific binding. Samples were analysed using a BD Accuri™ C6 Cytometer (Becton, Dickinson and Company BD). The flow cytometry data were analysed by using the FlowJo software or the C6 Accurì software.

### Statistical analysis of expression data

Three transcriptomic datasets from CLL samples analysed on Affymetrix HG-U133 Plus 2.0 chips (Herold et al., 2011; Chuang et al., 2012) were obtained and selected from a public database, Gene Expression Omnibus (GEO) at NCBI (National Centre For Biotechnology Information, https://www.ncbi.nlm.nih.gov/geo/). Statistical analyses have been carried out within the R environment (http://www.r-project.org). Dataset signals were preprocessed with the Robust Multi-Array Average (RMA) method available in Affy R package (https://www.rdocumentation.org/packages/affy/versions/1.50.0) obtaining background corrected, normalized, log transformed probe intensities. Bi-weight mid-correlation was applied to analyze expressions of studied genes by using the WGCNA package (Langfelder and Horvath, 2008) and the obtained correlation matrixes were used to draw clustered heatmaps with pheatmap package (https://cran.r-project.org/web/packages/pheatmap/index.html). For time to first treatment evaluation (TTFT), the same datasets were divided into two groups based on the median expression level of the gene of interest, whose expression distributions were evaluated with density function by stats R package. Kaplan-Meier curves were computed with Survival package (https://cran.r-project.org/web/packages/survival/index.html) and Survminer package (https://cran.r-project.org/web/packages/survminer/index.html) was used to plot them. Wilcox test was used to calculate *p*-values.

### Statistical methods

Comparison of ZNF224 expression between groups of patients was based on the Mann-Whitney U test performed by using the statistical platform R (R Foundation for Statistical Computing, Vienna, Austria). The non parametric Kuskall-Wallis ANOVA (followed by pairwise Mann-Whitney U test) was used as omnibus test in case of more than two groups. No adjustment was made for multiple comparisons. When appropriate, *t*-test was used to calculate statistical significance. The discrimination accuracy of ZNF224 expression was further explored using the Receiver Operating Characteristic (ROC) Curve. In this case, the corresponding Area under the Curve (AUC) with the 95% Confidence Intervalla (CI) was computed. The choice of the optimal cut-off value was based on the maximization of the Youden Index (i.e. sensitivity + specificity).

Differences were considered significant when *p* ≤ 0.05 (*) or highly significant when *p* ≤ 0.001 (**).

## Results

### ZNF224 expression increases during CLL progression

In a previous study, we found that ZNF224 mRNA expression levels were higher in CLL patients compared to healthy donors (Busiello et al., 2017). Here, to evaluate if ZNF224 could be an early molecular marker of disease progression and response to drug treatment we analyzed the expression levels of ZNF224 in B lymphocytes isolated from peripheral blood of 77 untreated CLL patients at different stages of disease and in a group of 28 treated patients (Supplementary Table S1).

Untreated patients were clustered in low-risk (Rai 0-Binet A, 36 patients), intermediate-risk (Rai I- Rai II -Binet B, 18 patients), and high-risk (Rai III-Rai IV-Binet C, 13 patients), according to Rai and Binet staging system classification (Rai et al., 1975; Binet et al., 1981; National Comprehensive Cancer Network (NCCN), 2017).

**FIGURE 1:**
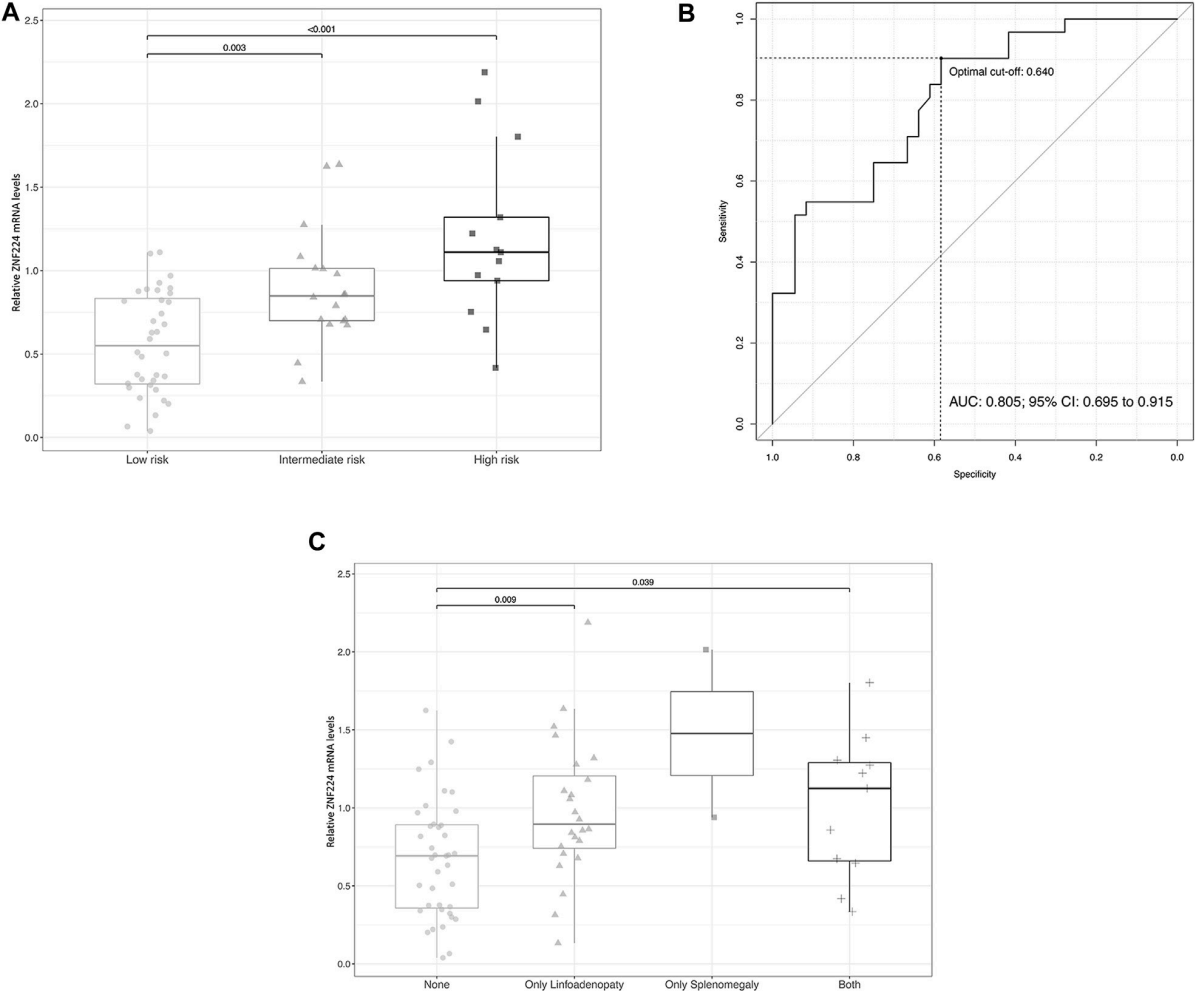
The expression levels of ZNF224 increase during CLL progression. **(A)** Evaluation of ZNF224 RNA expression in CLL patients stratified according to the risk of disease progression. **(B)** ROC curve showing accuracy of ZNF224 expression in differentiating medium and high-risk patients from low-risk patients. **(C)** Evaluation of ZNF224 RNA expression in CLL patients divided according to the presence of lymphadenopathy and/or splenomegaly.

Total RNA was purified from CD5^+^ B-cells and ZNF224 mRNA levels were determined by Real Time qRT-PCR analysis. We observed that the expression of ZNF224 progressively increased in intermediate (*p* = 0.003) and high-risk groups (*p* ≤ 0.001) with respect to low-risk (Figure 1A).

To further investigate if the ZNF224 expression can be used to discriminate between low (Rai 0-Binet A) and medium/high risk (Rai I-IV, Binet B/C) of progression in patients with CLL, receiver operating characteristic curve (ROC) was used. The corresponding AUC was equal to 0.805 (95% CI: 0.695–0.915) and the analysis suggested, as optimal cut-off, the value of 0.64 which allowed to obtain a sensitivity of 0.903 (95% CI: 0.774–1) and a specificity of 0.583 (95% CI: 0.417–0.750) (Figure 1B). Although this analysis should be performed on a larger number of patients, ZNF224 cut-off value could represent a possible boundary separating patients with a higher risk of disease progression with respect to others.

We also divided the patients according to the presence of lymphadenopathy and/or splenomegaly, typically associated with disease progression (Scarfò et al., 2016). Interestingly, we observed a significant increase in ZNF224 expression in patients with lymphadenopathy and both lymphadenopathy and splenomegaly (Figure 1C).

**FIGURE 2:**
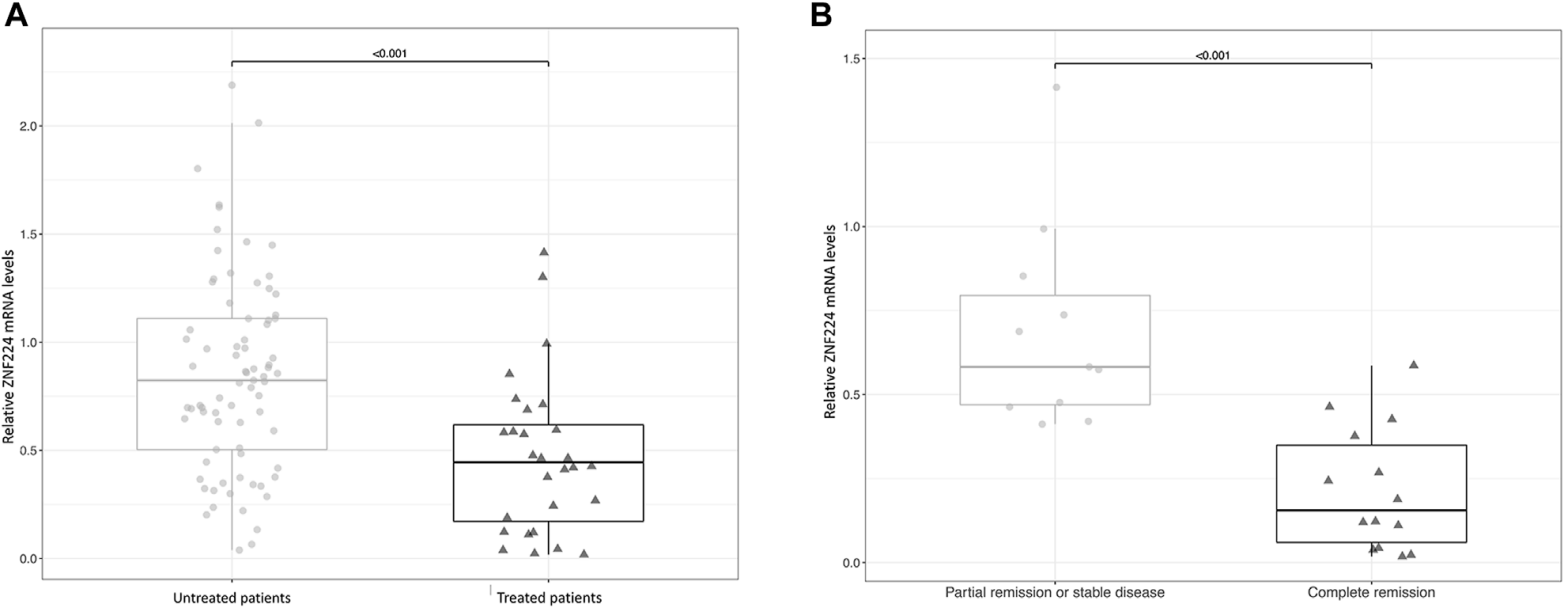
The expression of ZNF224 decreases in CLL patients after therapy. **(A)** Evaluation of ZNF224 RNA expression in untreated and treated CLL patients. **(B)** Evaluation of ZNF224 RNA expression in CLL patients receiving treatment and stratified into two groups according to their treatment outcome.

Then, to assess whether ZNF224 represents a marker of therapy responsiveness in CLL patients, we examined its expression levels in untreated and treated patients. As shown in Figure 2A, treatment resulted in a significant reduction of ZNF224 transcript. Subsequently, patients receiving treatment were classified into two groups according to their treatment outcome. One group was composed of patients undergoing treatment with kinase inhibitors and showing partial remission (5 patients) or stable disease (6 patients), the other group included patients which underwent chemotherapy ( ± combination with immunotherapy) and showing complete remission (14 patients) (Figure 2B). Interestingly, a dramatic reduction in ZNF224 levels was observed in patients in complete remission compared to patients showing partial remission or stable disease. This result suggests ZNF224 levels can be a powerful tool for evaluation of therapy responsiveness.

ZNF224 expression was investigated in relation to that of nine genes previously described as differently related to CLL disease. The subset includes CCND3, whose transcription activated by ZNF224 causes deregulated proliferation of CLL cells (Busiello et al., 2017), TCL1A and DDR1 both overexpressed in CLL and other hematological cancers and correlated with disease progression and adverse prognosis (Herling et al., 2009; Cader et al., 2013; Guo et al., 2019; Sidhu et al., 2021); in particular, TCL1A, augmented in most B-cell tumors, has been proposed as a potential predictive marker of outcome following treatment in CLL patients (Browning et al., 2007). ZAP-70 and CD38 are reported as reliable predictors of disease progression (Damle et al., 1999; Crespo et al., 2003) whereas LPL, ITGA4, MCL1 have been recently proposed as prognostic of negative outcome for patients with CLL (Browning et al., 2007; Van Bockstaele et al., 2007; Pepper et al., 2008; Mansouri et al., 2010). The subset also comprises CD5, which, although of uncertain prognostic significance, is indicated by various reports to contribute to CLL cell survival advantage, by inducing the production of anti-apoptotic cytokines (Garaud et al., 2011; Jaseb et al., 2019).

**FIGURE 3:**
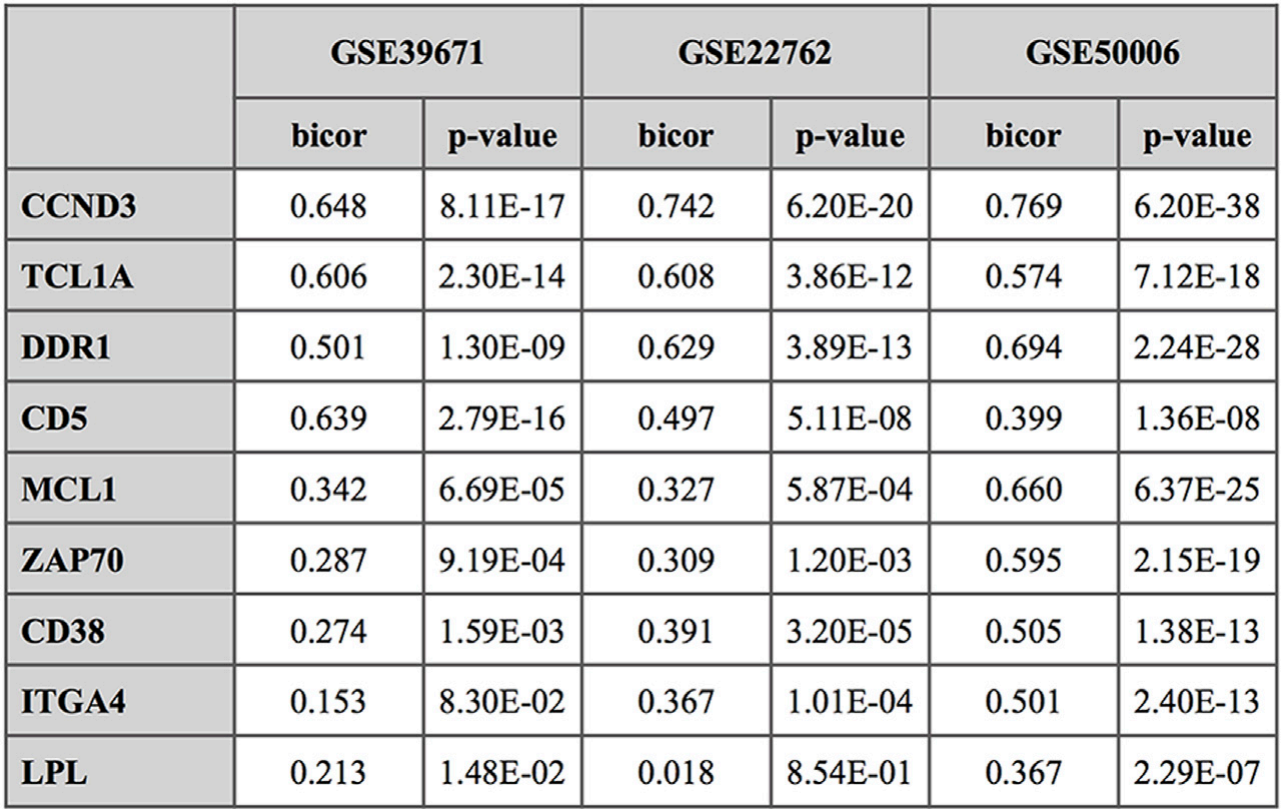
Correlation analysis of ZNF224 expression levels with selected CLL prognostic markers. The correlation is expressed in terms of bicor index (bi-weight mid-correlation); *p*-value for significance are also indicated.

Expression levels of each of these genes were related to that of ZNF224 by using datasets GSE39671, GSE22762, consisting of PBMC samples, and GSE50006 composed of B-cells samples, all of them available through NCBI Gene Expression Omnibus repository (http://www.ncbi.nlm.nih.gov/geo). Correlation indices (see methods) are reported in Figure 3 in terms of bicor and *p*-values. In the two PBMC datasets expression of ZNF224 is highly correlated (bicor≥0.5) with that of CCND3, TCL1A, DDR1 and CD5, as well as, but less strongly, related with the other genes in the subset (MCL1, ZAP70, CD38, ITGA4, LPL). In B-cells (GSE50006) ZNF224 expression is well related with all the genes of the subset, with bicor values above 0.5 for almost all of them.

**FIGURE 4:**
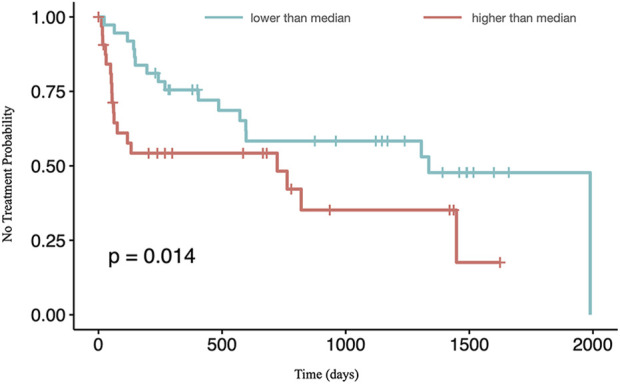
High ZNF224 expression correlates with a shorter Time To First Treatment (TTFT). Data from GSE22762 were stratified according to ZNF224 median expression value, with blue and red lines representing data respectively below and above the cut-off level.

Expression of ZNF224 in CLL patients was studied in relation to the evolution of the disease by using the time to first treatment (TTFT) data available in GEO dataset GSE22762. The results (Figure 4) show that high expression of ZNF224 is associated with shorter TTFT, indicating a possible negative impact of high ZNF224 expression levels on clinical evolution in CLL. The same analysis, performed on dataset GSE39671, is reported in Supplementary Figure.S1, where, although ZNF224 expression appears not significantly correlated with TTFT, a similar trend was observed.

The data obtained from evaluating ZNF224 levels in our cohort of patients and the strong positive correlation of ZNF224 expression with some CLL predictive and prognostic markers, obtained by bioinformatic analysis, lead us to speculate that ZNF224 could represent a potential marker of disease progression and therapy responsiveness in CLL patients.

### ZNF224 is involved in the activation of the NF-κB pathway

Previous literature data showed a ZNF224 involvement in the control of the NF-κB pathway in bladder carcinogenesis through the interaction with DEPDC1 and the subsequent reduction in the expression of the A20 gene, a negative regulator of NF-κB transcription factor (Harada et al., 2010).

The NF-κB signaling is constitutively activated in CLL patients and this activation is known to play an essential role in cancer initiation, progression, and resistance to treatment, through the modulation of a large number of proteins controlling proliferation and apoptosis (Furman et al., 2000; Endo et al., 2007).

**FIGURE 5:**
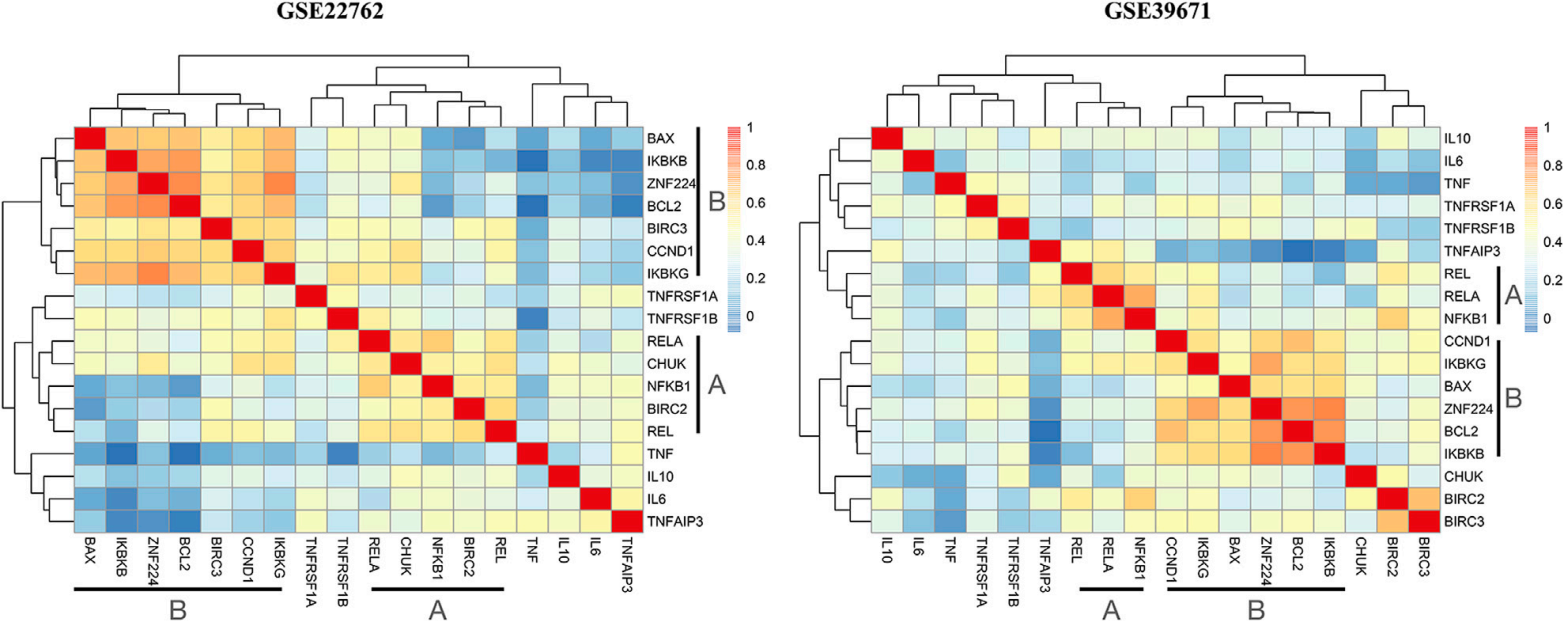
Expression of NF-kB pathway genes is related to ZNF224. Bi-weight mid-correlation was applied to analyze expressions of studied genes by using the WGCNA package and the obtained correlation matrixes were used to draw clustered heatmaps. A color gradient was used to represent the degree of correlation, with blue corresponding to correlation absent and red to maximum.

We hypothesized that ZNF224 exerted a proliferative and anti-apoptotic effect in CLL cells *via* the NF-κB pathway. To test such a hypothesis, firstly, we interrogated GSE39671 and GSE22762 datasets and analyzed the expression levels of a set of 17 genes, including genes involved in NF-κB signal transduction (IKBKG, IKBKB, CHUK, RELA, REL, NFKB1, TNFAIP3) or known to be regulated by NF-κB transcriptional activity (BCL2, CCND1, BAX, TNF, TNFRSF1A, TNFRSF1B, inhibitor of apoptosis BIRC2 and BIRC3, pro and anti-inflammatory cytokines IL6 and IL10) in relation to ZNF224 gene expression. For both datasets, clustered heatmaps are reported in Figure 5, while bicor indices and *p*-values for ZNF224 correlation with the genes of the subset are reported in Supplementary Figure.S2. Figure 4 shows that a small cluster (A), observed in both datasets, includes NF-κB1, REL, and RELA proteins, all of their components of the NF-kB complex and actually the predominant ones in CLL cells (Collart et al., 1990; Almaden et al., 2016). A larger cluster (B), showing many of the highest correlation levels, includes, in addition to ZNF224, other genes known to be involved in activating the pathway, such as IKBKB and IKBKG, coding for IKKβ and IKKγ, two proteins responsible for freeing NF-κB from the inhibitory block. The same cluster also includes CCND1 and BCL-2 family members BCL2 and BAX, all of them known targets of NF-κB transcriptional activation (Guttridge et al., 1999; Catz and Johnson, 2001; Grimm et al., 2005). These data confirm that elements of canonical NF-kB machinery are coordinately expressed in peripheral blood cells of CLL patients.

By looking at the correlation values reported in Supplementary Figure.S2, ZNF224 expression appears to be strongly related to several elements of the 17-gene panel. In addition to the already mentioned IKBKB and IKBKG, a weak but clear correlation was found with CHUK, the gene coding for IKKα, another component of the IKK complex that regulates the cascade of events leading to NF-κB signaling activation (Israël, 2010).

We also evaluated (Supplementary Figure.S3) BCL2/BAX ratio in both GSE22762 and GSE39671 datasets, obtaining values higher than 1, i.e. pro-proliferative and anti-apoptotic, for the large majority of patients (120/130 and 96/107 respectively).

Furthermore, we appreciated a significant positive correlation between ZNF224 expression levels and some molecular markers of CLL proliferation and aggressiveness in B lymphocytes from a small cohort of 22 CLL patients we analyzed (Supplementary Figure.S4).

**FIGURE 6:**
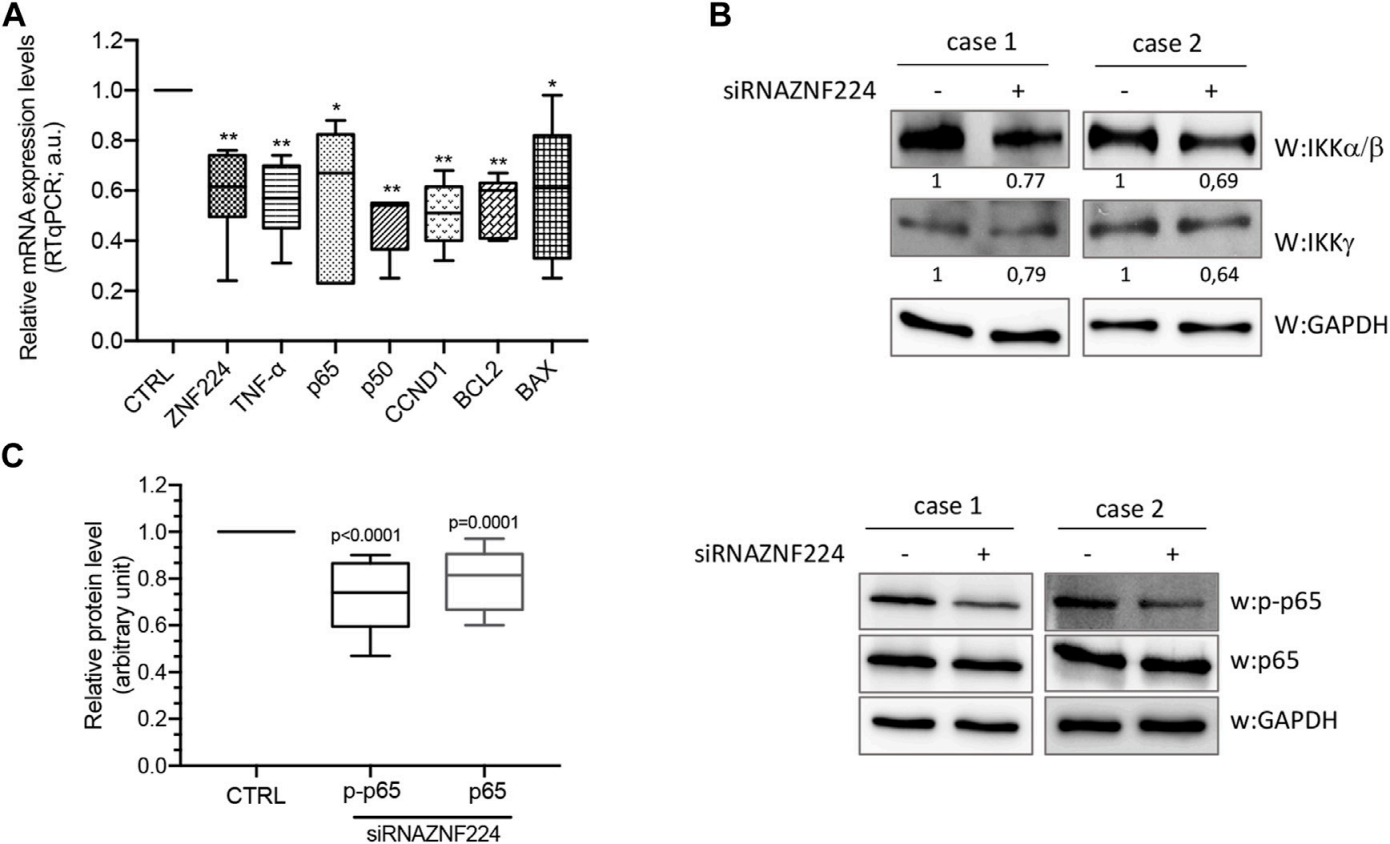
NF-kB pathway activation occurs by ZNF224. PBMCs of 12 CLL patients transfected with siRNAZNF224 or scramble siRNA were harvested 72 h after transfection for total protein extraction. **(A)** RTqPCR of NF-kB target genes shows their down-modulation following ZNF244 knock-down. **(B)** Western blot analysis of IKKα/β and IKKγ protein levels in two CLL PBMC samples silenced or not for ZNF224. **(C)** The histogram represents the relative protein expression levels of p-p65 and total p65 in 12 CLL PBMCs samples silenced for ZNF224 compared to control scramble siRNA (CTRL). Right panel shows two representative western blot analyses. GAPDH was used as control of loaded protein extracts.

The involvement of ZNF224 in the NF-κB pathway was investigated by evaluating the expression of several genes under NF-kB transcriptional control in CLL cells silenced for ZNF224. More in detail, PBMCs from 12 CLL patients were transfected with siRNA for ZNF224 or control scrambled siRNA and by RT-qPCR we showed the reduction of p50 and p65, components of the NF-κB transcription factor family, CCND1, BCL2 and BAX, targets of NF-κB involved in proliferation/survival signaling (Figure 6A). Interestingly, we also observed downmodulation of the pro-inflammatory cytokine TNF-α, whose high expression levels in sera of CLL patients have been found associated with aggressiveness and disease progression.

Besides, using western blot analysis we showed that ZNF224 knockdown negatively affected the catalytic subunits of IKK complex (IKKα, β and γ) in two CLL patients (Figure 6B). Then, we measured the phosphorylation levels of NF-κB p65 at Ser-536, a modification that promotes p65 transactivation and represents a critical event in the NF-κB signaling activation (Zhang et al., 2015). We found that ZNF224 knockdown caused a decrease in both phospho-p65 and total p65 levels (Figure 6C).

Altogether, these results suggest that ZNF224 sustains the activity of the canonical NF-κB pathway in CLL.

### ZNF224 knock-down enhances spontaneous and drug-induced apoptosis in CLL cells and inhibits cell proliferation

Subsequently, we evaluated the anti-apoptotic and proliferative effects of ZNF224 in PBMC from CLL patients silenced for ZNF224.

**FIGURE 7:**
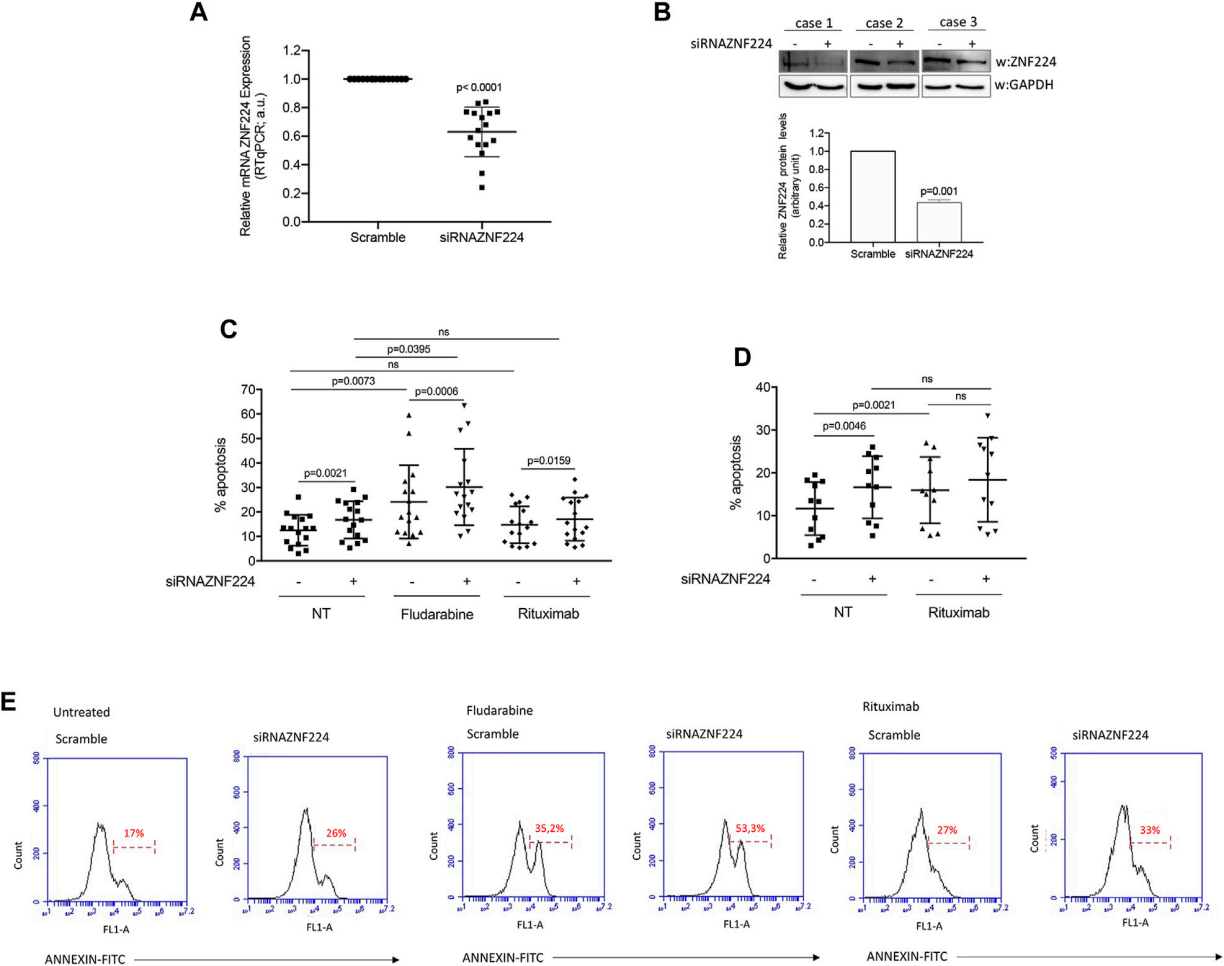
Effects of ZNF224 silencing on spontaneous and drug-induced apoptosis in CLL PBMCs. **(A)** ZNF224 expression in PBMC of 16 CLL patients silenced for ZNF224 was evaluated by RTqPCR. **(B)** Western blot analysis of ZNF224 protein levels in three CLL PBMC samples silenced or not for ZNF224. Densitometric analysis of ZNF224 protein levels is shown. **(C)** Histograms showing % apoptosis obtained from PBMCs of 16 CLL patients silenced for ZNF224 (+), untreated (NT) or treated with rituximab or fludarabine and compared to scramble CLL PBMCs (-), as control. **(D)** Histograms showing % apoptosis obtained from PBMCs of 11 CLL patients responsive to rituximab silenced ZNF224 (+), untreated (NT) or treated with rituximab and compared to scramble (-) CLL PBMCs, as control. **(E)** Representative flow cytometric analysis of apoptosis obtained from PBMCs of one CLL patient silenced for ZNF224 and scramble CLL PBMCs as control, untreated or treated with drugs.

PBMCs were purified from peripheral blood of 16 CLL patients with stable disease and transiently transfected with short interfering RNA (siRNA) targeting ZNF224 or with scramble siRNA as negative control. The silencing of ZNF224 was checked 72 h after transfection on RNA extracted from an aliquot of each CLL sample by qRT-PCR. In Figure 7A was shown the ZNF224-silencing, whose efficiency was variable among the different PBMCs from CLL patients. The reduction of ZNF224 expression was also evaluated at protein level in three CLL samples (Figure 7B).

In the meantime, the CLL samples were treated with 1 μM of the purine analog fludarabine or 10 μg/ml of the anti-CD-20 antibody rituximab or not subjected to any treatment and kept in culture for additional 48 h. Then, apoptosis was evaluated by Annexin V staining and flow cytometry. As shown in Figure 7C, 16 untreated samples silenced for ZNF224 showed a significant increase in spontaneous apoptosis compared to control scramble CLL cells (*p* = 0.0021). Fludarabine treatment significantly affected apoptosis of these CLL cells (*p* = 0.0073). Interestingly, we also observed sensitization to fludarabine-induced cell death in ZNF224-silenced cells (*p* = 0.0006), thus indicating that ZNF224 down-modulation could enhance the efficacy of chemotherapeutic agents in CLL patients. On the contrary, we did not observe a significant increase in apoptosis following rituximab treatment, due to the presence of five not responsive samples.

Therefore, in Figure 7D we reported the only 11 samples responsive to rituximab out of 16 treated (*p* = 0.0021), and we observed that ZNF224 knock-down did not enhance the rituximab-induced apoptosis in these samples. This result led us to speculate that silencing of ZNF224 and rituximab treatment could act on the same proliferative and anti-apoptotic pathways (Koivula et al., 2011). In accordance, it has been proved that rituximab reduced the phosphorylation of NF-κB-inducing kinase, diminished IKK kinase activity, and NF-κB DNA-binding activity (Jazirehi et al., 2005).

**FIGURE 8:**
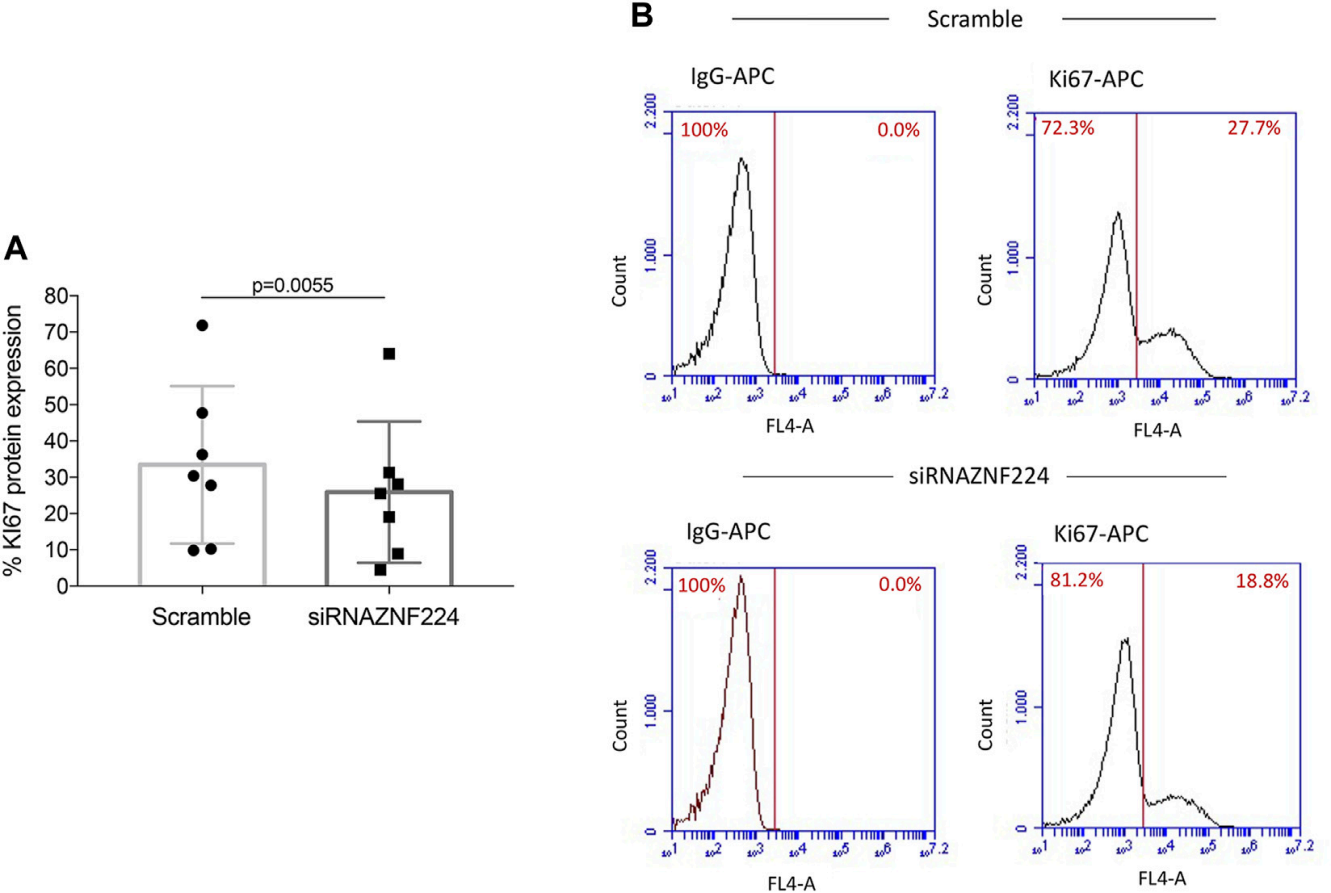
ZNF224 knock-down affects the proliferation of CLL PBMCs. Proliferation was evaluated by analyzing the Ki67-APC levels **(A)** Histogram of flow cytometric Ki67 assessment in PBMCs from seven CLL patients silenced for ZNF224 and scramble CLL PBMCs as control. **(B)** Representative flow cytometric analysis of Ki-67 expression in PBMCs from one CLL patient silenced for ZNF224 and scramble CLL PBMCs as control.

Finally, we also found that ZNF224 knock-down in PBMCs from seven CLL patients significantly reduced the expression of the proliferative marker Ki67 (Figure 8). This result, together with the above-shown downmodulation of CCD1 RNA (Figure 6A), indicates that ZNF224 silencing hindered cell proliferation.

## Discussion

Biological heterogeneity is one of the main causes of the variable clinical course of CLL, with survival ranging from months to decades.

Improving the management of this hematological malignancy requires a deeper understanding of its molecular pathogenesis and the identification of novel prognostic markers and therapeutic targets.

Increased proliferative ability of CLL cells and impaired apoptosis contribute to CLL progression and treatment resistance (Chiorazzi et al., 2005; Hillmen, 2011; Burger, 2020), although the mechanisms adopted by CLL cells to escape apoptosis and/or undergo deregulated proliferation, and the involved factors are not yet fully clarified (Kipps et al., 2017).

The zinc-finger transcription factor ZNF224 exerts an oncogenic role in CLL, preventing CLL cell lines from cell-cycle arrest and apoptosis (Busiello et al., 2017).

In this study, we used primary chronic lymphocytic leukemia cells to improve the comprehension of ZNF224 function in CLL in a more physiological setting, evaluating its relationship with disease progression and therapy responsiveness and investigating the molecular mechanism by which ZNF224 affects the delicate and crucial balance between apoptosis and proliferation, encouraging the latter.

We provide the first indication that ZNF224 could be a new potential biomarker of disease progression and therapy responsiveness, based on the relationship between ZNF224 expression and known prognostic and predictive markers and the assessment of its levels in clinical-risk stratified CLL patients (Rai et al., 1975; Binet et al., 1981; National Comprehensive Cancer Network (NCCN), 2017) and in treated patients classified according to their treatment outcome. The evaluation of ZNF224 expression levels, combined with established clinical and molecular markers, could contribute to more accurate risk stratification in CLL patients and provide an effective method for early monitoring of adverse events during therapy.

Accumulating evidence indicates that dysregulated KRAB-ZFPs take part in the regulation of crucial signaling pathways during tumorigenesis and cancer progression. As a typical member of this protein family, ZNF224 has been found involved in different oncogenic regulatory networks associated with cell survival and proliferation (Cesaro et al., 2017; Cesaro et al., 2021b; Sobocińska et al., 2021). Among them, ZNF224 plays a crucial role in the NF-kB pathway activation in bladder cancer and myeloma cells (Harada et al., 2010; Sun et al., 2014), thus exerting an oncogenic function through the activation of anti-apoptotic and pro-survival signals.

NF-kB has been recognized as a pathogenic factor in CLL and other hematological malignancies (Trombetti et al., 2021). Constitutive NF-kB activation promotes inhibition of apoptosis and resistance to chemotherapy. However, the mechanisms underlying NF-kB deregulation are not fully understood. Gene mutations leading to NF-kB activation are infrequent in CLL compared to other B-cell malignancies, while signals from the microenvironment and received through surface receptors, such as the B-cell receptor (BCR), appear to be involved in the activation of downstream cell survival pathways, including NF-kB (Chiorazzi et al., 2005; Mansouri et al., 2016).

Here, through bioinformatic analysis and *in vitro* assays in primary CLL cells, we provided evidence that ZNF224 expression could contributed to the regulation of NF-kB pathway. Indeed, we found a strong positive correlation between the expression values of ZNF224 and both genes involved in the activation of NF-kB pathway, such as IKBKG and IKBKB, and genes target of NF-kB, such as BCL2, CCND1 and BAX. Accordingly, knockdown of ZNF224 in CLL cells is accompanied by reduced activation of the NF-kB pathway and decreased RNA expression of NF-kB target genes.

High expression levels of BCL2 and BAX, despite its pro-apoptotic role, have been described in many CLL patients (Kitada et al., 1998; Aviram et al., 2000). It has been observed that the imbalance between pro-proliferative BCL2 and pro-apoptotic BAX expression, which leads to a high BCL2/BAX ratio in CLL cells, rather than expression levels of the individual proteins, is associated with improved survival and progression of the disease. Besides, BCL2/BAX ratio has been proposed as a prognostic marker associated with treatment response (Del Principe et al., 2016; Helaly et al., 2021).

The fact that ZNF224 expression in CLL patients is associated with BCL2/BAX ratios higher than 1 (Supplementary Figure.S3), as well as with high MCL1 levels (Figure 3), strengthens its proposed oncogenic role and predictive potential as a marker/indicator of aggressiveness and poor prognosis. Instead, absence of correlation was found between ZNF224 and TNFAIP3, the gene encoding protein A20, whose genetic alterations are not associated with the constitutive activation of NF-κB pathway in CLL (Frenzel et al., 2011; Philipp et al., 2011).

Moreover, the strong relationship between ZNF224, BCL2 and BAX in the analyzed datasets, and the downmodulation of BCL2 and BAX RNA following ZNF224 knockdown support the notion that overexpression of both anti- and pro-apoptotic proteins of the BCL-2 family occurs in CLL, contributing to dysregulated apoptosis (Kitada et al., 1998; Aviram et al., 2000; Helaly et al., 2021) and indicates the involvement of ZNF224 in the regulation of their expression. The molecular mechanisms that underlie this regulatory function of ZNF224 are worthy of clarification in the future.

The finding of ZNF224 as one of the factors enhancing NF-kB signaling may contribute to clarify the molecular mechanisms of NF-kB deregulation in CLL and to develop new strategies for targeting this pathway.

Moreover, we showed that ZNF224 silencing in CLL cells is accompanied by an increased both spontaneous and fludarabine-induced apoptosis and a reduced expression of the proliferative marker Ki67. Altogether, these data strongly indicate that persistent high expression levels of ZNF224 could trigger deregulated cell growth and apoptosis resistance in CLL, thus paving the way for the development of anti-ZNF224 therapeutics.

On the other hand, we cannot rule out that, as a zinc finger transcription factor, ZNF224 may regulate the expression of different target genes related to multiple signaling pathways controlling cell survival and proliferation in CLL. Global identification of ZNF224 target genes and interacting proteins will be required to improve understanding of its regulatory network in cancer.

## Data Availability

Publicly available datasets were analyzed in this study. This data can be found here: https://www.ncbi.nlm.nih.gov/geo.
