# Supplementary material for: Biological relevance of ZNF224 expression in chronic lymphocytic leukemia and its implication IN NF-kB pathway regulation
Source: Front Mol Biosci. 2022 Nov 9;9:1010984. doi: 10.3389/fmolb.2022.1010984 (PMC9681601; doi:10.3389/fmolb.2022.1010984)
Supplement: Supplementary file 1 [file DataSheet1.docx]

Supplementary Material


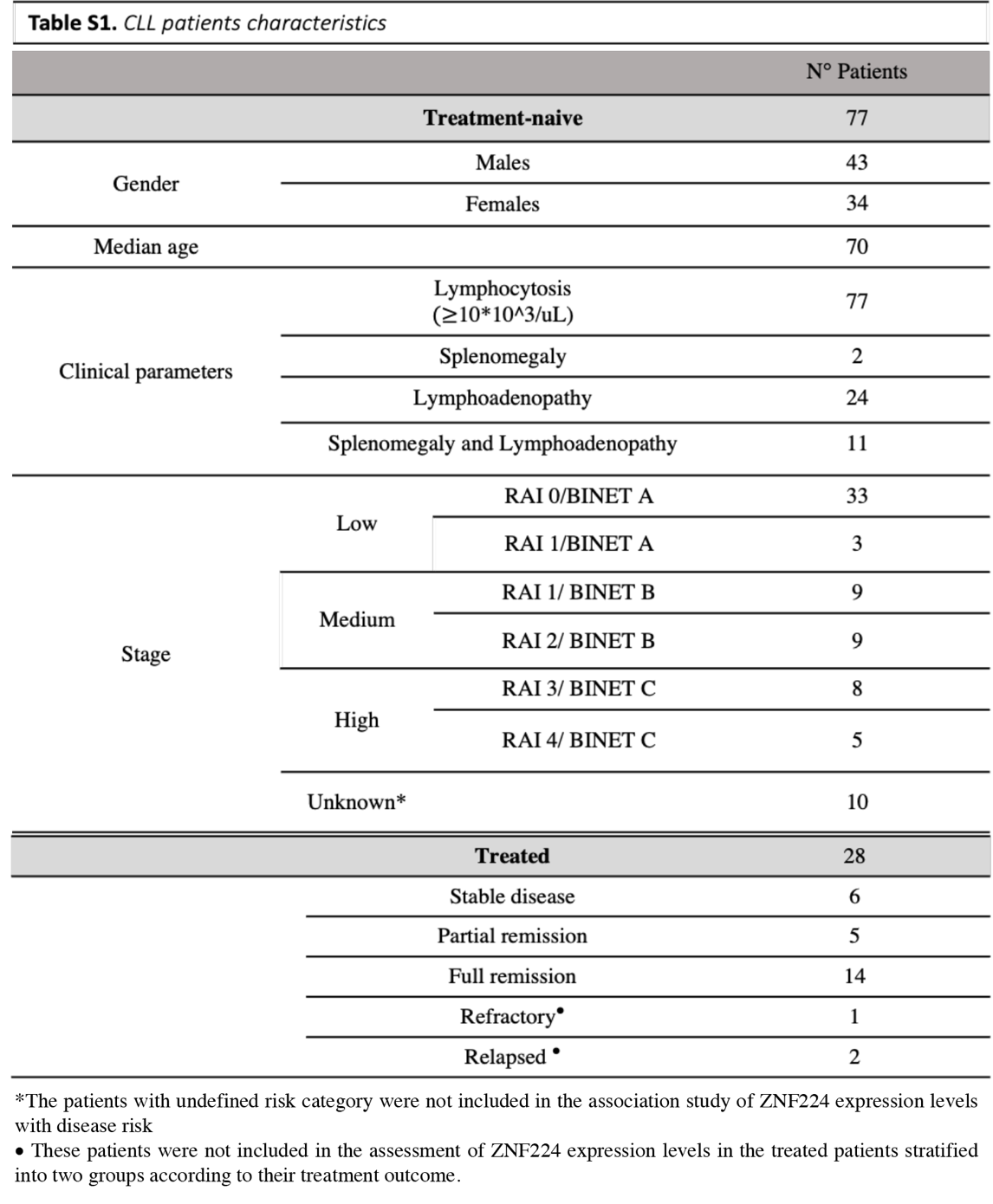


**Table S1: Characteristics of CLL patients.**


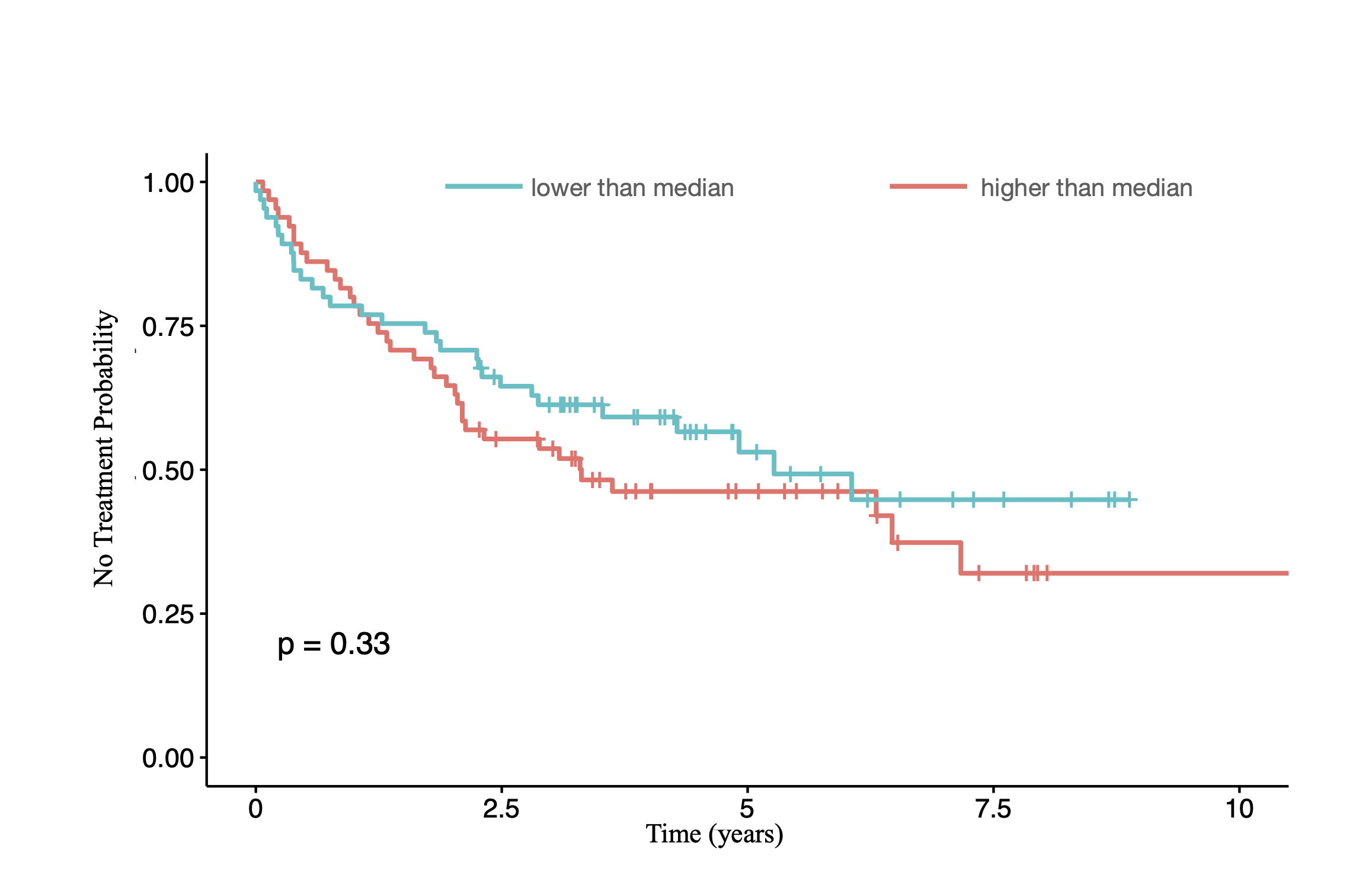


**Figure S1: Time To First Treatment analysis (TTFT) for GSE39671**. Data stratified according to ZNF224 median expression value, are divided along blue and red lines depending on whether they are below or above the cut-off level of ZNF224 expression.


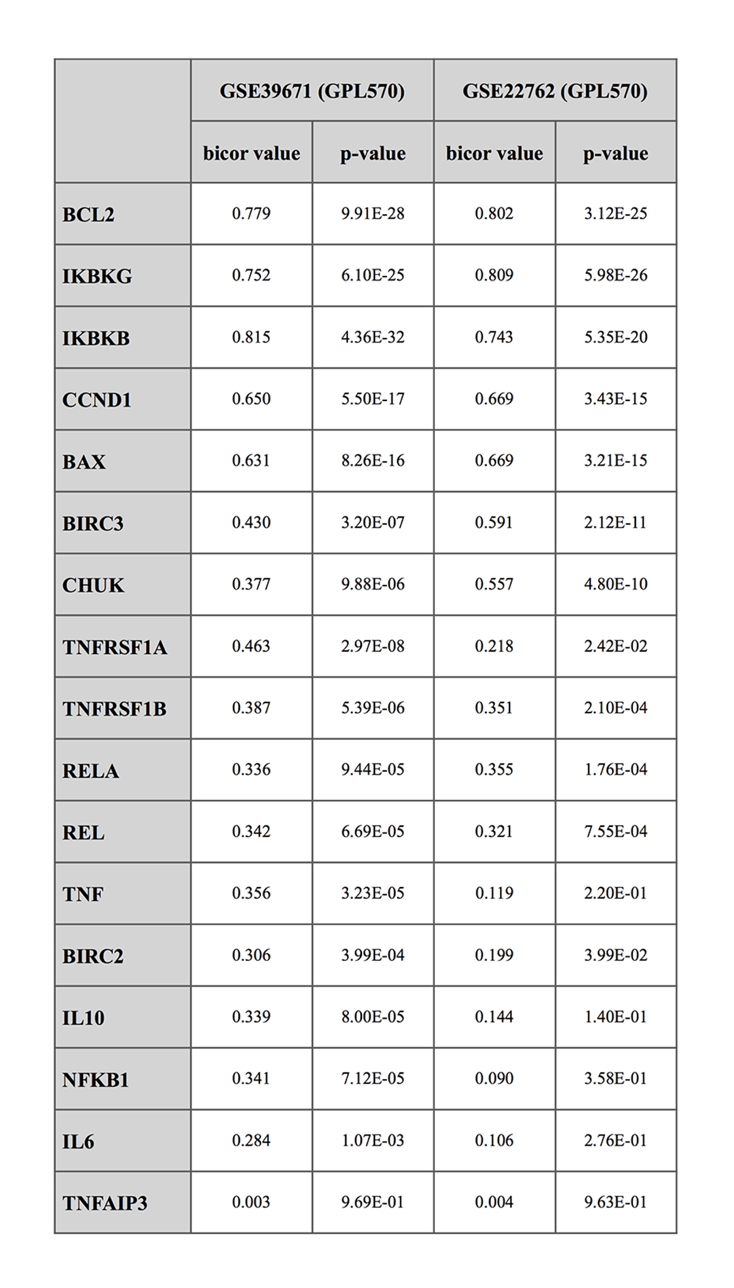


**Figure S2:** **Expression levels of ZNF224 were correlated with a set of 17 genes involved in NF-kB pathway.** The correlation is expressed as bicor index obtained from a bi-weight mid-correlation; p-values for significance are also indicated


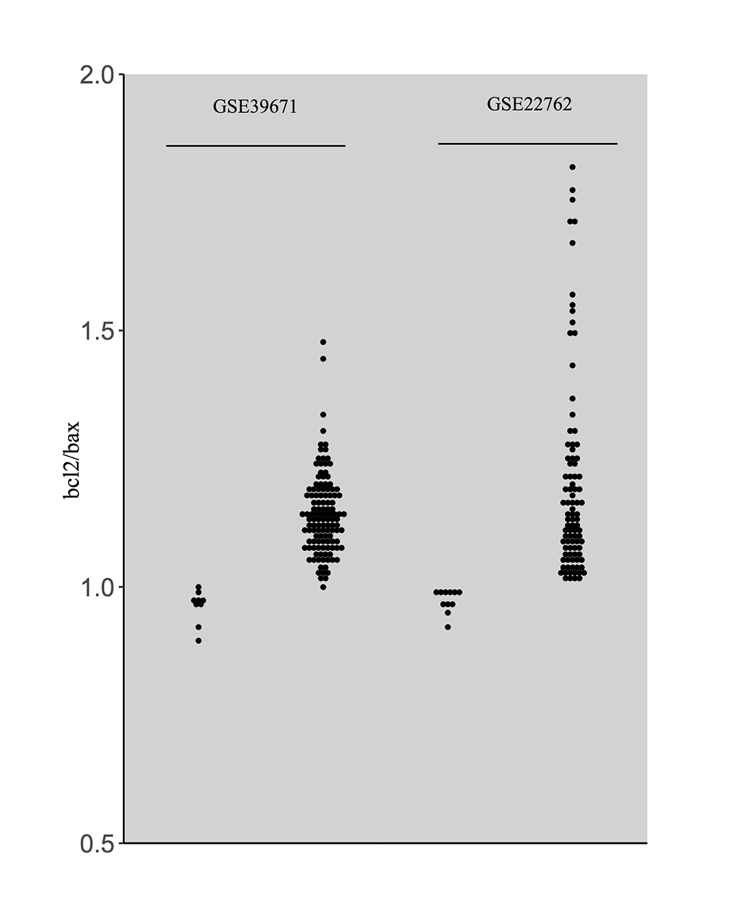


**Figure S3:** **BCL2/BAX ratio distribution on GSE39671 and GSE22762 datasets.** In GSE39671, 10/130 patients show bcl2/BAX ≤1, and 120/130 show bcl2/BAX >1; GSE22762 behaves similarly, with 11/107 patients with bcl2/BAX ≤1 and 96/107 having bcl2/BAX >1.


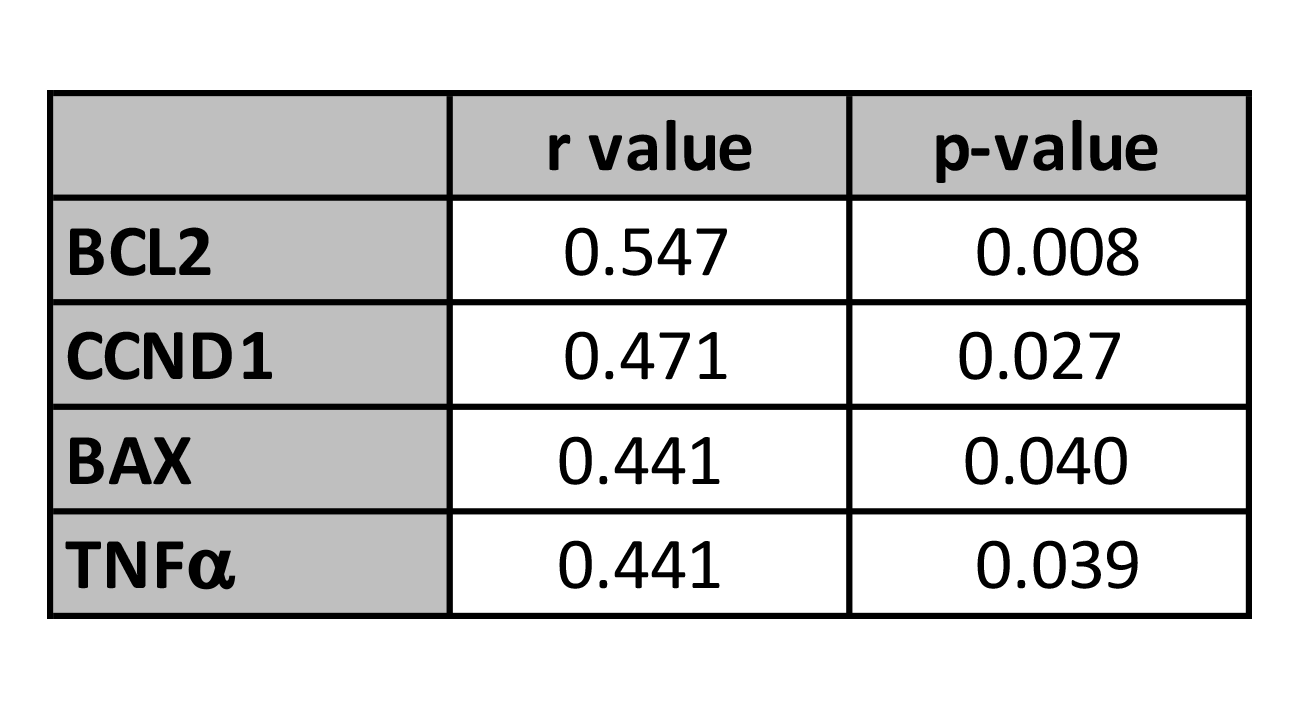


**Figure S4: Correlation analysis of ZNF224 expression with some molecular markers of CLL aggressiveness in a cohort of 22 CLL patients.** ZNF224 expression is positively related to BCL2, CCND1, BAX, and TNFα mRNA. Correlation was estimated using Spearman Correlation Coefficient (r value).
